# Serum Negative Chronic Autoimmune Thyroiditis (SN‐CAT): A Case Report of Three Siblings Without Familial History of Autoimmune Thyroid Diseases (ATDs)

**DOI:** 10.1002/ccr3.72455

**Published:** 2026-04-14

**Authors:** Sundus Huma, Nosheen Khan, Umar Khayam, Zmarak Ahmed Khan, Suleman Elahi Malik, Kamran Khan, Kamil Ahmad Kamil

**Affiliations:** ^1^ Department of Medicine Khyber Medical College Peshawar Pakistan; ^2^ Department of Internal Medicine Khyber Teaching Hospital Peshawar Pakistan; ^3^ Department of Endocrinology Khyber Teaching Hospital Peshawar Pakistan; ^4^ Department of Otorhinolaryngology (ENT) Hayatabad Medical Complex Peshawar Pakistan; ^5^ Department of Internal Medicine Mirwais Regional Hospital Kandahar Afghanistan

**Keywords:** autoimmune thyroid disease, case report, Hashimoto's thyroiditis, pediatric hypothyroidism, serum‐negative chronic autoimmune thyroiditis, sporadic thyroiditis

## Abstract

This case highlights the rare manifestation of a less prevalent form of chronic autoimmune thyroiditis, serum‐negative chronic autoimmune thyroiditis, within a sibling cluster without detectable autoantibodies and unclear genetic links, with no familial history, challenging current understanding of autoimmune thyroid disease. Ultrasound findings remain crucial for diagnosis, underscoring the need for clinical vigilance in atypical pediatric hypothyroidism.

## Graphical abstract

We report three siblings with antibody‐negative hypothyroidism and ultrasound features of autoimmune thyroiditis. Clinicians should consider serum negative chronic autoimmune thyroiditis (SN‐CAT) in pediatric hypothyroidism despite negative antibodies, particularly when imaging and biochemical findings are consistent, to avoid delayed diagnosis and management.

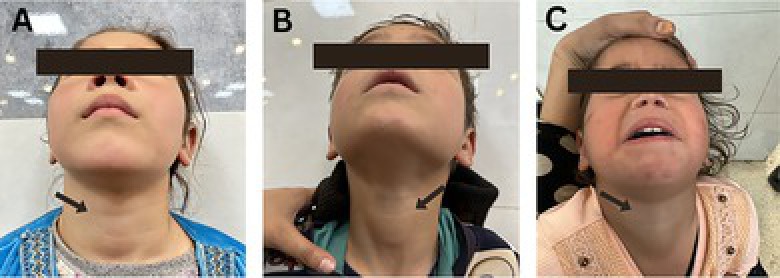

## 1 Introduction

Chronic autoimmune thyroiditis (CAT), also known as Hashimoto's thyroiditis (HT), is the most frequent autoimmune thyroid disorder (ATD) and the most common cause of hypothyroidism in children and adolescents, characterized by autoimmune destruction of the thyroid gland. The most significant features of HT include atrophy of follicular cells, lymphocytic infiltration, goiter, and fibrosis [1, 2]. The ultimate diagnosis of HT is based on a combination of clinical, serological, and imaging features. These include the presence of antibodies against the thyroid gland, antithyroperoxidase (anti‐TPO), and anti‐thyroglobulin (anti‐TG), which demonstrate high diagnostic sensitivity, with positivity rates of approximately 86.3% and 84.3%, respectively, in patients with HT [3]. Ultrasonography typically reveals a hypoechogenic and dyshomogeneous thyroid parenchyma, while biochemical evaluation often shows elevated thyroid‐stimulating hormone (TSH) levels accompanied by normal or low serum thyroid hormone concentrations. The majority of patients (around 75%) are euthyroid at the time of diagnosis, whereas the remaining minority present with subclinical or overt hypothyroidism [4].

About 90% of patients with CAT present with serum positive for antibodies, which is the classical HT, while the remaining 10% can present with no detectable antibodies against the thyroid gland, known as serum‐negative chronic autoimmune thyroiditis (SN‐CAT) [5].

SN‐CAT is recognized as a distinct clinical entity, yet large scale studies remain limited despite its common clinical occurrence. In 1988, Baker et al. reported the first case of SN‐CAT [6]. While epidemiologic information is insufficient, the prevalence of the condition is estimated to be approximately 5% of individuals presenting with hypothyroidism [7]. Patients with SN‐CAT exhibit symptoms similar to those of classical HT presentation, including a painless neck lump, lymphocytic infiltration, and signs of hypothyroidism. However, studies have shown that SN‐CAT is milder than HT [8]. Diagnosis of SN‐CAT is made exclusively through ultrasound and thyroid function tests (TFTs) [9]. SN‐CAT, similar to classic HT, is more prevalent in females, which supports an autoimmune etiology of SN‐CAT.

SN‐CAT may have biological and technical explanations, but evidence for these mechanisms is limited. One reason for the failure of immunoassay tests to detect antibodies could be epitope variability, as some noncanonical, conformational, or transient antibodies may escape detection. Additionally, serum factors may mask epitopes, causing interference with binding [10]. Another possible mechanism involves thyroid antigens generating isoform‐specific sequences through alternative splicing; assays that utilize canonical proteins may miss antibodies to these spliced variants. Moreover, in progressing diseases, epitope spreading can enlarge the antibody repertoire beyond narrow antigen panels, potentially resulting in missed later‐stage or atypical autoantibodies [11]. Furthermore, immune exhaustion resulting from chronic self‐antigen stimulation can dampen B‐cell responses, limiting differentiation into antibody‐secreting cells and causing tissue damage even in the absence of detectable autoantibodies [12]. Technical assessment limitations also contribute; methods like hemagglutination require higher titers and may overlook low‐titer or nonagglutinating antibodies, whereas more sensitive techniques such as radioimmunoassays can detect a broader range [13]. Moreover, increased iodine intake has been associated with autoimmune thyroiditis, promoting thyroid‐directed immune activation even without circulating autoantibodies [14]. Lastly, infectious triggers may cause local or transient thyroid immune activation, but they typically do not lead to the production of serum autoantibodies [15]. Overall, both biological variability and technical limitations may contribute to seronegativity, although the exact mechanisms remain incompletely understood.

With limited literature available, it is evident that similar to classic CAT, which has a familial prevalence of 43.59%, individuals with SN‐CAT often exhibit a genetic predisposition or a positive family history, with environmental factors accounting for the remaining etiology [8, 16].

We report a case of three siblings with a similar clinical presentation of SN‐CAT.

## 2 Case Presentation

A family presented to the Endocrinology Division, MTI Khyber Teaching Hospital (KTH) with three siblings: a 7‐year‐old female, a 5‐year‐old male, and a 3‐year‐old female. All three have been previously diagnosed with hypothyroidism and are currently receiving treatment. There was no family history of hypothyroidism or other autoimmune diseases among parents or first‐degree relatives.

A comparative overview of their clinical, biochemical, and radiological characteristics is summarized in Table 1. While the clinical course and key diagnostic events for the three siblings are outlined in Table 2.

**TABLE 1:** Clinical, biochemical, and radiological characteristics of the three siblings diagnosed with serum‐negative chronic autoimmune thyroiditis (SN‐CAT).

Parameter	Case 1 (5‐year‐old male)	Case 2 (7‐year‐old female)	Case 3 (3‐year‐old female)
Age at dagnosis	2 years	7 years	3 years
Symptoms	Anterior neck swelling, growth retardation, developmental delay	Mild anterior neck swelling, growth retardation	Mild anterior neck swelling
Family history	Negative	Negative	Negative
Physical examination	Visible goiter, no focal lesions	Mild neck swelling, no other abnormalities	Mild neck swelling, no focal lesions
TSH level (mIU/L) (Reference range: Age 1–6 years: 0.7–5.97 mIU/L Age 7–11 years: 0.6–4.84 mIU/L)	> 100	> 150	> 100
FT4 Level (pmol/L) (Reference range: Age 1–12 years: 10–22 pmol/L)	< 5	< 2	< 2
Anti‐TPO & Anti‐TG antibodies	Negative	Negative	Negative
Neck ultrasound findings	Diffuse thyroid enlargement with heterogeneous parenchymal echotexture, generalized hypoechogenicity, and internal septations	Diffusely enlarged thyroid gland with preserved echogenicity and no focal nodules	Diffusely enlarged thyroid gland with preserved echogenicity and no focal nodules
Color Doppler	Mildly increased vascularity	Normal vascularity	Normal vascularity
Diagnosis	SN‐CAT	SN‐CAT	SN‐CAT
Treatment	Levothyroxine 6 mcg/kg/day PO	Levothyroxine 6 mcg/kg/day PO	Levothyroxine 6 mcg/kg/day PO

*Note:* This table summarizes the demographic details, presenting symptoms, physical examination findings, thyroid function tests, antibody status, ultrasound and Doppler characteristics, final diagnosis, and initial treatment of all three siblings. Despite the absence of thyroid autoantibodies and a negative family history, each child demonstrated hypothyroidism and ultrasound features consistent with SN‐CAT.

Abbreviations: Anti‐TG, anti‐thyro globulin; Anti‐TPO, antithyroid‐peroxidase; FT4, free thyroxine; PO, per os; TSH, thyroid stimulating hormone.

**TABLE 2:** Clinical timeline of diagnostic evaluation, management, and follow‐up outcomes of the three siblings diagnosed with serum‐negative chronic autoimmune thyroiditis (SN‐CAT).

Timeline	Clinical event	Investigation	Outcome
Age 2 years	Male sibling (Case1) presented with neck swelling and developmental delay	TFTs	Hypothyroidism diagnosed
Initial evaluation	Thyroid ultrasound	Diffuse enlargement	Suspicion of autoimmune thyroiditis
Screening of siblings	Elder (Case 2) and younger (Case 3) siblings evaluated	TFTs	Hypothyroidism confirmed
Antibody testing	Anti‐TPO and Anti‐TG	Negative	SN‐CAT suspected
Treatment initiation	Levothyroxine started	—	Symptom improvement
Follow‐up	Regular endocrinology visits every 3 months	TFT monitoring	Stable thyroid function

*Note:* This table outlines the chronological sequence of clinical presentation, diagnostic evaluation, laboratory and radiological investigations, treatment initiation, and follow‐up outcomes in all three siblings. It highlights the progression from initial symptom recognition to diagnosis and ongoing management, demonstrating consistent clinical improvement and stabilization of thyroid function with levothyroxine therapy despite the absence of detectable thyroid autoantibodies.

Abbreviations: Anti‐TG, anti‐thyro globulin; Anti‐TPO, antithyroid peroxidase; TFTs, thyroid function tests.

**FIGURE 1:**
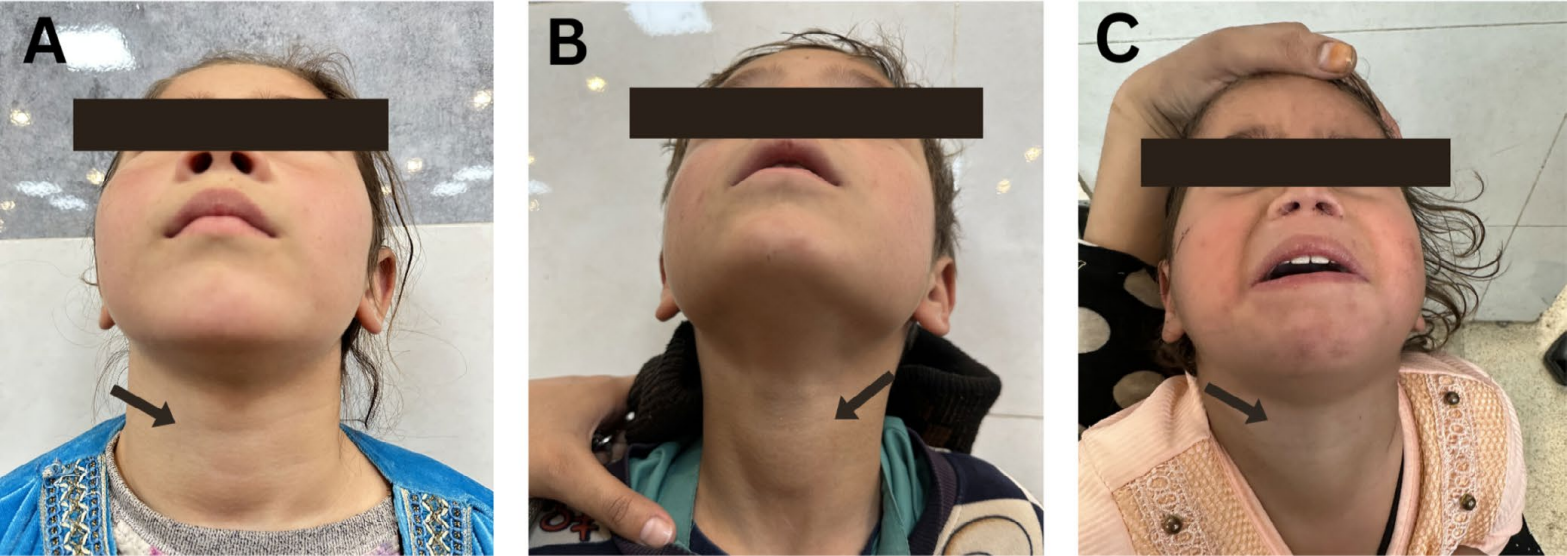
Clinical presentation of anterior neck swelling in all three siblings diagnosed with Serum‐Negative Chronic Autoimmune Thyroiditis (SN‐CAT). The 7‐year‐old female sibling presented with visible anterior neck swelling at her initial endocrinology visit following her brother's diagnosis. (B) The 5‐year‐old male sibling referred by a general practitioner for progressive anterior neck swelling and growth retardation. (C) The 3‐year‐old female sibling who was evaluated after both older siblings were diagnosed with hypothyroidism, demonstrating mild anterior neck swelling. Arrows indicate the area of thyroid enlargement in each child.

Clinical photographs demonstrating anterior neck swelling in all three siblings are shown in Figure 1.

## 3 Diagnosis, Management, and Follow‐up Outcomes

Given the absence of familial hypothyroidism, further testing was conducted. Anti‐TPO and anti‐TG antibodies were negative in all three siblings; therefore, based on clinical, biochemical, and radiological findings, the diagnosis of SN‐CAT was established.

Standard therapy with levothyroxine was initiated for each child and follow‐up was planned. During follow‐up, all three siblings demonstrated clinical improvement with stabilization of TFTs under levothyroxine therapy. Follow‐up thyroid ultrasound showed near‐normal echogenicity and vascularity of the thyroid gland. The patients continue to undergo regular endocrinology follow‐up every 3 months.

## 4 Discussion

This case report describes three instances of SN‐CAT within a sibling cluster, highlighting significant variations in clinical presentation, ultrasound findings, and the progression of the disease. Despite the absence of a familial history of thyroid or any other autoimmune disorders, the presence of SN‐CAT in all three siblings, coupled with the male child exhibiting a more severe form in terms of clinical symptoms of the disease than his female siblings, underscores the atypical nature of this case.

SN‐CAT is a variant of HT characterized by the absence of detectable thyroid autoantibodies [8]. Compared to classical HT, SN‐CAT generally presents with a milder clinical picture, with lower TSH levels, higher FT4 levels, and smaller thyroid volumes [8]. However, in the present cases, all three siblings demonstrated markedly elevated TSH and significantly reduced FT4 levels, suggesting severe biochemical hypothyroidism at presentation. This discrepancy may reflect delayed diagnosis or variability in the clinical spectrum of SN‐CAT in pediatric populations. Additionally, limited data is reported on such cases in the literature, with no exclusive case reports or discussions on SN‐CAT involving family members. Only a small number of cases comparing the clinical features of SN‐CAT with classical HT have been published. However, in 2020, a case series of 49 patients with SN‐CAT was studied in a cross‐sectional analysis [17]. The close variations between classical HT and SN‐CAT raise questions about the exact prevalence of the disease. There is a need for case series on such instances of SN‐CAT to trace their variations and clarify the actual prevalence.

The diagnosis of SN‐CAT remains challenging, relying predominantly on the presence of hypoechoic thyroid changes on ultrasound alongside negative thyroid antibody results [18]. In evaluating antibody‐negative presentations of ATDs, further investigations are often recommended to exclude alternative etiologies and potential diagnostic errors. These may include repeat thyroid antibody assays to account for laboratory variability, assessment of other autoimmune markers such as antinuclear antibodies (ANA) or celiac disease‐related antibodies, evaluation of iodine status, and, in selected cases, genetic testing for thyroid peroxidase (TPO) gene mutations [19]. In the present cases, iodine status was assessed and found to be within the normal range, thus making iodine‐related thyroid dysfunction unlikely. However, advanced genetic testing such as TPO mutation analysis was not available in the local clinical setting, which represented a limitation of the diagnostic workup. Thyroid antibody testing was performed using standard immunoassay techniques; while repeated evaluations could further reduce the possibility of laboratory‐related seronegativity, the combination of consistent clinical features, markedly abnormal thyroid function tests, and characteristic ultrasound findings supported the diagnosis of SN‐CAT in these children. Following the initiation of levothyroxine therapy and consistent monitoring, the ultrasound findings gradually normalized in echogenicity and vascularity, while T3 and T4 levels remained within the therapeutic range, underscoring the significance of ongoing management in such cases.

Familial clustering of CAT, particularly HT, is well established, with studies demonstrating higher prevalence among female relatives and frequent detection of thyroid autoantibodies in siblings of affected individuals [20, 21, 22]. However, the sibling cluster in this case presents a unique deviation from the typical familial pattern. All three siblings were affected despite the absence of a known family history or detectable autoantibodies, and the male child exhibited a more severe clinical presentation and markedly deranged TFTs compared to his female siblings. This atypical severity in the male child contrasts with the usual female predominance observed in ATDs, highlighting the unusual nature of this presentation and raising significant questions about the underlying pathophysiology that must be explored.

Similar cases in the literature have reported familial presentations of ATDs. One report detailed two siblings, one diagnosed with Graves' disease and the other with HT, supporting the concept of genetic predisposition to a spectrum of ATDs [23]. Another case involved siblings with opposing thyroid conditions: One had transient neonatal hyperthyroidism, while the other had hypothyroidism, both attributed to maternal autoimmune thyroiditis [24]. Although many reports highlight familial clustering and genetic predisposition in the overall population of CAT, there is no such case describing SN‐CAT in multiple siblings without any clear genetic predisposition.

The autoimmune etiology of SN‐CAT is supported by its clinical similarities to classic HT as well as its initial discovery alongside Type 2 diabetes and rheumatoid arthritis, despite the consistent absence of detectable autoantibodies [25, 26, 27, 28]. Genetic predisposition, environmental factors, and epigenetic influences are commonly implicated in ATDs [17], yet the precise pathophysiology of SN‐CAT remains uncertain. This case series is noteworthy for its sporadic nature, underscoring the need for further research into nongenetic triggers and the underlying mechanisms contributing to SN‐CAT.

## 5 Conclusion

The unusual presentation of the SN‐CAT within a sibling cluster, as discussed, raises several intriguing questions regarding its prevalence, etiology, and pathophysiology. Given the absence of a familial link or associated antibodies, new avenues are opened up for a deeper investigation. Clinicians should therefore consider SN‐CAT in pediatric patients presenting with hypothyroidism even in the absence of detectable thyroid antibodies, particularly when ultrasound findings support autoimmune thyroid involvement. Further research is essential to explore the genetic, environmental, and immunological factors that may contribute to this phenomenon, which could lead to improved diagnostic criteria and management strategies for similar cases in the future.

## Author Contributions

**Sundus Huma:** conceptualization, data curation, methodology, resources, supervision, writing – original draft. **Nosheen Khan:** conceptualization, methodology, resources, writing – original draft. **Umar Khayam:** data curation, formal analysis, investigation, visualization, writing – original draft. **Zmarak Ahmed Khan:** data curation, investigation, methodology, project administration, writing – review and editing. **Suleman Elahi Malik:** project administration, supervision, writing – review and editing. **Kamran Khan:** formal analysis, project administration, writing – review and editing. **Kamil Ahmad Kamil:** project administration, supervision.

## Funding

The authors have nothing to report.

## Ethics Statement

Case reports require written informed consent from the patient, which has been obtained. However, case reports do not require ethical review from the institutional review committee at our institution.

## Consent

Written informed consent was obtained from the patient for the publication of this case report and images. A copy of the written consent is available for review by the Editor‐in‐Chief of this journal upon request.

## Conflicts of Interest

The authors declare no conflicts of interest.

## Data Availability

The authors have nothing to report.
